# Pharmacological Properties of Ginsenoside Re

**DOI:** 10.3389/fphar.2022.754191

**Published:** 2022-04-06

**Authors:** Xiao-Yan Gao, Guan-Cheng Liu, Jian-Xiu Zhang, Ling-He Wang, Chang Xu, Zi-An Yan, Ao Wang, Yi-Fei Su, Jung-Joon Lee, Guang-Chun Piao, Hai-Dan Yuan

**Affiliations:** ^1^ College of Pharmacy, Yanbian University, Jilin, China; ^2^ College of Integration Science, Yanbian University, Jilin, China; ^3^ Key Laboratory of Natural Medicines of the Changbai Mountain, Ministry of Education, Yanbian University, Jilin, China

**Keywords:** ginsenoside Re, pharmacological activities, pharmacokinetics, toxicology, bioactive component

## Abstract

Ginsenoside Re is a protopanaxatriol-type saponin extracted from the berry, leaf, stem, flower bud, and root of *Panax ginseng*. In recent years, ginsenoside Re (Re) has been attracting attention as a dietary phytochemical. In this review, studies on Re were compiled by searching a combination of keywords, namely “pharmacology,” “pharmacokinetics,” and “toxicology,” in the Google Scholar, NCBI, PubMed, and Web of Science databases. The aim of this review was to provide an exhaustive overview of the pharmacological activities, pharmacokinetics, and toxicity of Re, focusing on clinical evidence that has shown effectiveness in specific diseases, such as diabetes mellitus, nervous system diseases, inflammation, cardiovascular disease, and cancer. Re is also known to eliminate virus, enhance the immune response, improve osteoporosis, improve skin barrier function, enhance intracellular anti-oxidant actions, regulate cholesterol metabolism, alleviate allergic responses, increase sperm motility, reduce erectile dysfunction, promote cyclic growth of hair follicles, and reduce gastrointestinal motility dysfunction. Furthermore, this review provides data on pharmacokinetic parameters and toxicological factors to examine the safety profile of Re. Such data will provide a theoretical basis and reference for Re-related studies and future applications.

## Introduction

Ginseng is a perennial herb belonging to the family Araliaceae and genus *Panax (P.)*. The plant has been used as a tonic in Chinese traditional medicine for more than 2000 years. It is also extensively used as a medicinal supplement across Asia and America (Jiang et al., 2020; Xu et al., 2020). *P. ginseng* Meyer (Asian ginseng), *P. quinquefolium* L. (American ginseng), and *Eleutherococcus senticosus* (Siberian ginseng) are the most common types of ginseng (Kiefer and Pantuso, 2003). All of these species are in the Araliaceae plant family. Extensive preclinical and clinical evidence in scientific literature support the significant beneficial effects of P. ginseng and P. quinquefolius L. in significant central nervous system, metabolic, infectious, and neoplastic diseases (Mancuso and Santangelo, 2017). Active components of most *P. ginseng* species include ginsenoside, polysaccharide, peptide, polyacetylenic alcohol and fatty acids (Dong et al., 2017). Of the active components, ginsenoside (i.e., ginseng saponin or triterpene saponin) is an important component responsible for many biochemical and pharmacological properties of the herb (Gillis, 1997). Currently, more than 30 natural ginsenosides have been extracted and their chemical structures have been identified. The main active ginsenosides are categorized into two groups based on the types of aglycone. The 20(*S*)-protopanaxadiol group includes ginsenosides Rb1, Rb2, Rb3, Rc, Rd, Rh2, compound K, and Rg3, and the 20(*S*)-protopanaxatriol group (PPT) comprises ginsenosides Re, Rf, Rg1, Rg2, and Rh1 (Ma et al., 2005). Of these, Re (C48H82O18, PubChem CID: 441921) is a major component (0.15%) of *P. ginseng.* We chose Re in the present study because of its high concentration in a number of commercially available *P. ginseng* extracts (Harkey et al., 2001). This water-soluble compound (Xie et al., 2005b) accounts for 23% of total saponins and is abundant in the leaves, stems, flower buds, berries, and roots of the plant (Joo et al., 2010; Bae et al., 2012; Kim et al., 2009). Previous research has shown that Re is more abundant in leaves, berries, and flower buds than in roots, and that it is the major saponin in *P. ginseng* fruits (Attele et al., 2002; Xie et al., 2004; Su et al., 2014). The percentage weight of Re extracts from American *P. ginseng* were 4.79, 3.5, and 0.4% in leaves, berries, and roots, respectively (Xie et al., 2005a; Han et al., 2012). This work showed that *P. ginseng* leaves and berries had the highest Re concentration, and that Re is the major ginsenoside in *P. ginseng* leaves. These findings also revealed that the Re content is different in various parts of the *P. ginseng* plant. In recent years, Re has been attracting attention as a dietary phytochemical, likely attributed to advantages such as ease of availability, low cost, high efficacy, straightforward isolation and purification techniques, and low side effects and toxicity risks (Quan et al., 2012). Re is a white crystalline powder that is readily soluble in methanol and ethanol. Its chemical properties include; melting point: 201–203°C; boiling point: 1011.8 ± 65.0°C; density: 1.38 ± 0.1 g/cm^3^; and acidity coefficient: 12.85 ± 0.70 (https://www.chemicalbook.com/ProductChemicalPropertiesCB5210824.htm). Previous research revealed *in vivo* and *in vitro* mechanisms that mediated diverse pharmacological activities of Re. Re has anti-diabetic (Table 1), neuroregulatory (Table 2), anti-inflammatory (Table 3), pro-cardiac (Table 4), anti-cancer (Table 5), anti-viral, anti-fungal and anti-oxidant effects. It is also known to improve skin barrier function, regulate cholesterol metabolism, alleviate allergic responses, enhance the immune response, improve osteoporosis, increase sperm motility, reduce erectile dysfunction, promote cyclic growth of hair follicles, and reduce gastrointestinal motility dysfunction (Table 6). In this review, the pharmacological actions and associated molecular mechanisms, pharmacokinetic characteristics, and toxicology of Re were summarized after researching major online databases. This review also describes the limitations of Re.

**TABLE 1:** Summary of anti-diabetes effects of Re.

Inducer	Experimental Model	Outcome and Proposed Mechanism	Reference(s)
	C57BL/6J ob/ob mice	FBG↑, IPGTT↑	Attele et al. (2002)
	C57BL/6J ob/ob mice	BG↑, FBG↑	Xie et al. (2005a)
HFD	Wistar rats	IR↑, GLUT4↑	Han et al. (2012)
HFD, GPL	C57BL/6J mice, HepG2 cells	p-LKB1↑, p-AMPK↑, SHP↓, SREBP1c↓, FAS↓, SCD1↓	Quan et al. (2012)
HFD, DII	Wistar rats, 3T3-L1 adipocytes	Glucose uptake↑, p-IRS-1↑, p-PI3K↑, Akt/PKCγ/λ↑, p-JNK↓, NF-κB↓	Zhang et al. (2008)
HSHF; HSHF+AM; HSHF+STZ	Wistar rats	BG↓, TC↓, TG↓, Lp-a↓, VEGF↓, IL-6↓, p-p38↓,insulin levles↑, HDL-C↑	Shi et al. (2016)
STZ	SD rats	BG↓, MDA↓, TC↓, TG↓, GSH↑	Cho et al. (2006)
STZ	SD rats	FBG↓, TNF-α↓, MDA↓, GSH↑	Liu et al. (2012)
HFD	C57BL/6 mice	TG↓, TC↓, LDL-C↓, GOT↓, GPT↓, MDA↓, p-JNK↓, p-IRS↓, p-tau↓, BG↑, HDL-C↑, Ach↑, GSH↑, SOD↑	Kim et al. (2017)
HFD	C57BL/6 mice	FG↓, TG↓, TC↓, LDL-C↓, AChE↓, MDA↓	Park et al. (2015)
DII	3T3-L1 cells	Glucose uptake↑, GLUT4↑, IRS-1↑, PI3K↑	Lee et al. (2011)
DII	3T3-L1 cells	TNF-α↓,TG↑, Glucose uptake↑, PPARγ-2↑, ap2↑, IRS-1↑, GLUT4↑, Adiponectin↑	Gao et al. (2013)
High glucose	RF/6A cells	LDH↓, MDA↓, p-Akt↓,ROS↑, CAT↑, GSH-Px↑, HIF-1α↑, Caspase-3↑, VEGF↑, Caspase-9↑	Xie et al. (2020)

**TABLE 2:** Summary of nervous system disease effects of Re.

Inducer	Experimental Model	Outcome and Proposed Mechanism	Reference(s)
Surgery	SD rats, Schwann cell	PCNA↑, GAP-43↑, S100↑, p-ERK1/2↓, p-JNK1/2↓	Wang et al. (2015)
MCAO model	SD rats	SOD↑, GSH-Px↑, Average microviscosity↓, MDA↓	Zhou et al. (2006)
MCAO model	SD rats	H^+^-ATPase activity↑, MDA↓	Chen et al. (2008)
TMT	IL-6(−/+) C57BL/6 mice	c-FOS-IR↑, IL-6↑, p-Akt↑, IFN-γ↓, TNF-α↓, IL-1β↓, MDA↓, ROS↓	Tu et al. (2017)
PCP	C57BL/6mice, GPx-1 knockout mice	GPx-1↑, PHOX activity↑	Tran et al. (2017)
RIS	SD rats	BDNF↑, Behavioral deficits↓, TH↓	Lee I et al. (2012)
CRS	C57BL/6J mice	BDNF↑, Nrf2↑, HO-1↑, SYP↑, PSD95↑, NLRP3↓, ASC↓, Caspase-1↓	Wang et al. (2021)
MPTP	C57BL mice	Bcl-2↑, iNOS↑, caspase-3↑, TH-positive neurons↑, Bax↓	Xu et al. (2005)
MA	PKCδ(+/−) C57BL/6 mice	SOD↑, catalase↑, GPx↑, DA↑, dopaminergic degeneration↓, PKCδ↓	Shin et al. (2014)
MA	DYN KO mice	κ-opioid receptor↓, P-mediated NK1 receptor↓	Dang et al. (2018)
CCl_4_	Primary dopaminergic cell	Neurites of TH cells↑, Neuritic lengths↓	Zhang et al. (2016)
MA	SH-SY5Y cell	Cell viability↑, GPx↑, GSH↑, TH activity↑, PKCδ↓	Nam et al. (2015)
	Dopaminergic neuronal cell, Hsp60 KD cell, PINK1 null dopaminergic cell lines	Hsp90↑, LRPPRC↑, Hsp60↑	Kim et al. (2012)
Rotenone	SH-SY5Y cells	SOD↑, GSH/GSSG↑, aconitase↑, Nrf2↑, ROS↓, Caspase-3↓, Bax/Bcl2↓, Cytochrome c↓	Gonzalez-Burgos et al. (2017)
6-OHDA	SH-SY5Y cells	Cell viability↑, GPX4↑, p-Akt↑, p-ERK↑, LDH↓, ROS↓, lipid peroxidation↓	Lee et al. (2020)
Scopolamine	CR mice, Wistar rats	Escape latency↓	Wang et al. (2010)
	Tg2576 mice	Aβ-40↓, Aβ-42↓	Zhou et al. (2020)
	CHO 2B7 cells, Aβ-lesioned mice	Aβ-40↓, Aβ-42↓	Chen et al. (2006)
Aβ-25-35 peptide	Kunming mice	phenylalanine↓, tryptophan↑, hexadecasphinganine↑, phytosphingosine↑, LPCs↑	Li et al. (2018)
Surgery and microdialysis	SD rats	DA↑, Ach↑, mPFC	Shi et al. (2013)
	N2a/APP695 cells	PPARγ↑, Aβ1-40↓, Aβ1-42↓, β-amyloid, BACE1↓	Cao et al. (2016)
Aβ+serum free	PC12 cells	LDH↓, cell toxicity↓	Ji et al. (2006)
Aβ	SH-SY5Y cells	GSH↑, SOD↑, GPx↑, ROS↓, Bcl2/Bax↓, Nrf2↓, Caspase-3/9↓, Cytochrome c↓, p-ASK-1↓, p-JNK↓, HO-1↓	Liu et al. (2019)
	Neuro-2a cells	MAP-2↑, p75↑, p21↑, TrkA↑, ChAT/VAChT↑	Kim M et al. (2014)

**TABLE 3:** Summary of anti-inflammation effects of Re.

Inducer	Experimental Model	Outcome and Proposed Mechanism	Reference(s)
C48/80, LPS	HMC-1 cell, A549 cell	Histamine secretion↓, IL-1α↓, IL-8↓, IL-10↓, RANTES↓	Bae et al. (2012)
TPA	BALB/c mice, Raw 264.7 cells	NO↓, MDA↓, ear edema↓, inflammatory cell infiltration↓, IL-1β↓, TNF-α↓	Paul et al. (2012)
LPS	SD rats, BALB/c mice, RAW264.7 cells	WBCs↑, neutrophil counts↑, TNF-α↓, IL-1β↓, IL-6↓, COX-2↓, iNOS↓, NO production↓, PGE2↓	Su et al. (2015)
LPS, TNBS	ICR mice	ZO-1↑, claudin-1↑, occludin↑, IL-1β↓, TNF-α↓, COX-2↓, iNOS↓, IL-6↓, colon shortening↓	Lee J et al. (2012)
LPS	C57BL/6 mice	ERs↑, PI3K/Akt↑, INF-γ↓, MCP-1↓, LDH↓, CK↓, AST↓, TNF-α↓, IL-1β↓, IL-6↓, p-p65↓, MAPKs↓	Chen et al. (2016)
LPS	ICR mice, A549, MH-S cells	Neutrophil↓, macrophage infiltration↓, NF-κB↓, MAPKs↓, c-Fos↓	Lee et al. (2018)
LPS	N9 microglia cells	NO↓, TNF-α↓, NF-κB↓, p-ERK↓, p-JNK↓, p-jun↓, p-IκB-α↓	Wu et al. (2007))
LPS	BV2 microglial cells	Cell viability↑, iNOS↓, COX-2↓, p-P38↓	Lee K et al. (2012)
LPS	RAW264.7 cells and primary rat hepatocytes	TNF-α↓, IL-6↓, PGE2↓, NO secreation↓, MAPKs↓, NF-κB↓	Quan et al. (2019)
TNF-α	EAhy926, HEK 293 cells	Cell viability↑, LDH↓, IL-6↓, p-IKK/IKK↓, p-IκB↓, p-NF-κB↓	Li Z et al. (2016)

**TABLE 4:** Summary of cardiovascular disease effects of Re.

Inducer	Experimental Model	Outcome and Proposed Mechanism	Reference(s)
I/R	SD rats	Haemodynamic change↑, [Ca^2 +^]_i_↓	Kim et al. (2011)
	Cardiomyocytes, Guinea pig ventricular myocytes	I(Ks) ↑, I(Ca,L) ↓	Bai et al. (2003), Bai et al. (2004)
LADCA ligation	Wistar rats, SD rat	Infarct size↓, MPO↓, PMN infiltration↓, ICAM-1↓	Jing et al. (2010), Li et al. (2013)
I/R	SD rats	Hemodynamic parameter↑, QRS complex↓, QT interval↓, R-R interval↓, TNF-α↓	Lim et al. (2013)
Isoproterenol	Wistar rats	TGF-β↓, p-Smad3↓, collagen I↓	Wang et al. (2019)
MI	SD rats	Heart rate↑, LVEF↑, LVPWd↑, LVPWs↑, IVSTd↑, IVSTs↑, SOD↑, FAK↑, PI3K↑, Akt↑, AMPKα↑, LVDd↓, LVDs↓, EDV↓, ESV↓, CK-MB↓, cTnT↓, MDA↓, Ang II↓, ANP↓, BNP↓, TGF-β1↓, Smad↓	Yu et al. (2020)
tBHP, MI/R	H9c2 cells, SD rats	miR-30c-5p↑, Apoptosis↓, LDH↓, p53↓	Wang et al. (2020)
GD	H9c2 cells	Cell viability↑, SOD↑, ATP depletion↑, LC3B-2↑, MDA↓	Zhang et al. (2020)
H/R	HL-1 cells	Cell viability↑, ATP Levels↑, LC3B-2↑, p-AMPK↑	Sun et al. (2020)
	Cat and human cardiomyocytes	[Ca^2+^]_i_ transient amplitude↑, Sarcoplasmic reticulum Ca^2+^ content↓	Wang et al. (2008b)
	Guinea pig ventricular myocytes	IKs↑, eNOS↑, PI3K↑, Akt↑	Furukawa et al. (2006)
	VSMCs	KCa↑, eNOS↑, PI3K↑, Akt↑	Nakaya et al. (2007)
	HUVEC	[Ca^2+^]_i_↑, NO↑, eNOS↑	Leung et al. (2007)
	HCAEC	Outward currents↑, SKCa currents↑	Sukrittanon et al. (2014)
Balloon	SD rats	vessel lumen↑, NO↑, cGMP↑, eNOS↑, PCNA positive cells↓	Gao et al. (2018)
PDGF-BB	VSMCs	cGMP↑, NO↑, p-eNOS/eNOS↑, p21↑, PCNA↓, cyclin D1↓, CDK4↓	Gao et al. (2019)
H_2_O_2_	HUVECs	NO↑, eNOS↑, SOD↑, GSH-Px↑, LDH↓, MDA↓	Huang et al. (2016)
Ox-LDL	HUVECs	ERα↑, PI3K↑, PKB↑, LOX-1↓, NADPH oxidase↓, NF-κB↓, p-p38↓	Yang et al. (2018)
bFGF	HUVECs, Wistar rats	Cell proliferation↑, hemoglobin content in ECMs↑, migration, tube formation↑, neo-collagen regenerate↑	Huang et al. (2005)
bFGF, Matrigel	HUVECs, C57/BL6 mice	Cell proliferation and migration↑, tube formation↑, neo-vessels density↓	Yu et al. (2007)

**TABLE 5:** Summary anti-cancer effects of Re.

Inducer	Experimental Model	Outcome and Proposed Mechanism	Reference(s)
CDDP	LLC-PK1 cells, Wistar rats	Cell viability↑, DPPH radical-scavenging activity↑, Caspase-3↑, Renal cortex tissue tubular damage↓	Lee W et al. (2012),
			Kim J et al. (2014)
CDDP	ICR mice	CAT↑, GSH↑, Bcl2/Bax↑, CRE↓, BUN↓, MDA↓, 4-HNE↓, CYP2E1↓, COX-2↓, iNOS	Wang et al. (2018c)
CTX	BALB/c mice	Erythropoietin↑, thrombopoietin↑, TPO↑, RBCs↑, hemoglobin↑, platelets S phase↑, Bcl-2↑, WBCs↓, thymus index↓, BMNC↓, spleen index↓, Bax↓, Caspase-3↓	Han et al. (2019)
	SW480 cells	Apoptosis↑, Cell proliferation↓	Xie et al. (2011)
	293T, MCF-7, A375, HepG2 cells	LDH release↑, Cell viability↓, ROS↓, Caspase-3↓	Yao et al. (2018)

**TABLE 6:** Summary of other disease effects of Re.

Effect	Experimental Model	Outcome and Proposed Mechanism	Reference(s)
Anti-viral	CVB3, and HRV3 infection HeLa and Vero cells	Cytotoxicity↓	Song et al. (2014)
Anti-viral and immune response	RV-induced ICR mice	Splenocyte proliferative↑, IL-4↑, IL-10↑, IL-12↑, IFN-γ↑, CD^4+^ cells↓, CD^8+^ cells↓	Su et al. (2014)
	H3N2-induced ICR mice	Th1↑, Th2↑	Song et al. (2010)
Anti-viral	Avian influenza H9N2 infected HUVEC cells	miR-15b↑, Cell viability↑, IP-10↓, DNA damage↓	Chan et al. (2011)
Immune response	CD^4+^ T cells	Cell viability↑, IFN-γ↓, IL-13↓, IRGM↓	Son et al. (2010)
	OVA-induced ICR mice	Th1↑, Th2↑	Sun et al. (2006)
Osteoblast differentiation	RANKL-induced Zebrafish	ERK↓, TRAP↓, cathepsin K↓	Feng and McDonald, (2011)
	MC3T3-E1 cells and Zebrafish model	ALP↑, Runx2↑, Colla1↑, Alp↑, Ocn↑	Park et al. (2016)
Against UVB radiation	UVB-induced HaCaT keratinocytes	GSH↑, SOD↑, ROS↓, MMP-2↓, MMP-9↓	Kim et al. (2016)
Improve skin barrier function	HaCaT keratinocytes	Filaggrin↑, Cornified envelope formation↑, Caspase-14↑	Shin et al. (2018)
Anti-oxidant	HaCaT keratinocytes	GSH↑, SOD↑, ROS, MMP-2↓, MMP-9↓	Oh et al. (2016)
	H_2_O_2_-induced *E.coli*	Fpg↑, ROS↓	Lim et al. (2016)
	H_2_O_2_ or ATA-induced chick cardiomyocytes	Cell viability↑, DCF fluorescence↓	Lee B et al. (2012)
Regulating Cholesterol Metabolism	High cholesterol-induced Wistar rats	CYP8B1↑	Kawase et al. (2014)
Alleviating allergic response	Histamine-induced ICR mice	IL-4↓, TNF-α↓, NF-κB↓, c-jun↓	Jang et al. (2012)
Increasing sperm motility	Fertile volunteer, Asthenozoospermic infertile patients	iNOS↑, NO↑	Zhang et al. (2006)
Restoring erectile dysfunction	Ethanol-induced SD rats	Nitrite↑, cGMP↑, ICP↑	Pyo et al. (20I6)
Promoting cyclic growth of hair follicles	Immunodeficient mice, C57BL/6 mice, HeLa cells	Hair shaft growth↑, P-Smad 2/3↑, p-FAK↑, p-ERK↑, p-JNK↑, TGF-β↓, SAMD↓	Li et al. (20I6)
Reducing gastrointestinal motility dysfunction	CP SD rats, DP SD rats	p-MLC20↑, MLCK↓, NO↑, adrenaline↑	Xiong et al. (2014)
	Cajal interstitial cells	Amplitude↓, frequency↓, cGMP↑	Hong et al. (2015)
	C48/80-induced Wistar rats	Hexosamine↑, adherent mucus↑, TBARS↓, XO↓, MPO↓, Bax↓, Bcl2↑	Lee et al. (2014)

## Pharmacokinetics of Re

Pharmacokinetic studies are necessary for observing and predicting the actions and interactions of drugs and for determining their efficacy and toxicity. The pharmacokinetics of Re have been studied in both animals and humans (Table 7), with major parameters, such as maximum concentration (T_max_), T_1/2_, and bioavailability examined. However, there is still little known about its metabolic and pharmacokinetic profiles.

**TABLE 7:** The main pharmacokinetic parameters of Re.

Route Adminstration	Dose	Model	Parameters											Reference
			AUC _(0-t)_ (ng/ml·h)	AUC _(0-∞)_ (ng/ml·h)	T½ (h)	T_max_ (h)	C_max_ (ng/ml)	MRT (h)	V_d_ (L/kg)	CL (L/h/kg)	RC	f (%)	F (%)	
i.v.	1 mg/kg	ICR mice (♀)	638.8 ± 197.0	639.3 ± 196.8	0.2 ± 0.03	—	—	0.2 ± 0.07	0.3 ± 0.2	1.7 ± 0.7	—	—	—	Joo et al. (2010)
	1 mg/kg	ICR mice (♂)	1437.6 ± 271.2	1442.0 ± 271.0	0.5 ± 0.08	—	—	0.5 ± 0.08	0.2 ± 0.07	0.7 ± 0.11	—	—	—	
p.o.	10 mg/kg	ICR mice	—	17.7 ± 4.5	—	0.4 ± 0.2	29 ± 25.4	0.76 ± 0.20	—	—	—	—	0.28	
	50 mg/kg		—	61.5 ± 37.0	—	0.7 ± 0.7	35 ± 4.3	2.0 ± 1.2	—	—	—	—	0.19	
p.o.	200 mg	Healthy volunteers	2.476 ± 2.281	2.699 ± 2.284	1.82 ± 0.75	1.19 ± 0.44	0.939 ± 0.549	—	—	124.054 ± 84.725	—	—	—	Liu et al. (2011)
i.v.	152.91 mg/kg	Rabbits	—	—	0.83	—	—	—	0.246	—	0.61	17	—	Chen et al. (1980)
i.p.					1.165				—		0.72	18	35	
s.c.	12.5 mg/kg	SD rats	2.771	2.963	2.399	1	0.56	—	—	—	—	—	—	Shi et al. (2013)
	25 mg/kg		6.328	8.073	2.531	1	2.19	—	—	—	—	—	—	
	50 mg/kg		12.630	14.295	2.157	1	3.72	—	—	—	—	—	—	
p.o.	200 mg/kg	SD rats	9,896.68 ± 1,234.48	11,830.85 ± 2,366.47	8.343 ± 6.148	0.9 ± 0.22	1,703.85 ± 104.15	14.924 ± 5.205	250.73 ± 159.7	0.32 ± 0.044	—	—	—	Chen et al. (2017)
p.o.	800 mg/kg XSTDT	SD rats	6 × 10^5^ ± 1 × 10^5^	6 × 10^5^ ± 1 × 10^5^	6 ± 3	6 ± 1	6 × 10^4^ ± 2 × 10^4^	8.6 ± 2.2	12.9 ± 3.5	1.45 ± 0.58	—	—	—	Dai et al. (2016)
p.o.	600 mg/kg QXSBP	SD rats	823.15 ± 97.94	958.34 ± 157.26	1.71 ± 0.39	0.56 ± 0.10	412.35 ± 89.16	—	—	—	—	—	—	Chen et al. (2021)
	60 mg/kg QXSBP		1,764.19 ± 265.38	1,906.79 ± 239.45	1.32 ± 0.38	0.50 ± 0.16	867.69 ± 103.29							
i.v.	5 ml/kg GGSQ	SD rats	2.16 × 10^6^ ± 0.59 × 10^6^	2.24 × 10^6^ ± 0.76 × 10^6^	2.25 ± 0.84	—	—	1.4 ± 0.65	39.08 ± 5.21	—	—	—	—	Ji et al. (2017)
i.v.	7.2 ml/kg SFI	SD rats	639.70 ± 134.61	653.77 ± 121.07	0.14 ± 0.03	—	3176.44 ± 515.91	0.18 ± 0.03	0.29 ± 0.04	1.48 ± 0.28	—	—	—	Shen et al. (2021)

### Absorption and Distribution

The time for saponins to reach T_max_ in rat plasma was less than 2 h, indicating that saponins are rapidly absorbed and readily distributed in tissues (Li et al., 2006; Gui et al., 2007). In humans, Liu et al. (2011) reported that the T_max_ of Re was 1.19 ± 0.44 h after oral ingestion. Another study showed that the T_max_ of Re was 0.75 h after oral administration of total *P.* notoginsenoside powder in rats, suggesting rapid absorption of Re in the gastrointestinal tract. The absolute bioavailability of Re was 7.06% (Li et al., 2006). Joo et al. (2010) revealed that the T_max_ of Re was 0.4 ± 0.2 h in ICR mice. The same study also showed that the oral bioavailability was 0.19–0.28%, suggesting that the absorption rate of Re was lower after oral administration. Shi et al. (2013) demonstrated that Re (12.5, 25 and 50 mg/kg, s.c. injection) was rapidly distributed to the cerebrospinal fluid and exhibited linear pharmacokinetics in rats, and that the T_max_ of Re was 1 h for all doses. However, for the lowest dose of 12.5 mg/kg, Re was not detectable in dialysates after 4 h. Extensive gastrointestinal metabolism, poor membrane permeability, and low solubility of deglycosylated products may limit the absorption of ginsenosides in the intestines. Therefore, the dose of test compounds must be high to detect ginsenoside content in plasma (Qi et al., 2011).

### Metabolism and Biotransformation

According to preclinical trials, several types of saponins, including ginsenosides Rg2, Rh1, F1, Rg1, and protopanaxatriol, may be metabolites of Re in human plasma and urine samples (Liu et al., 2011). After administration of Re (200 mg/kg, p.o. for 24 h), the major excreted ginsenoside metabolites in rat urine included Rg1 and Re. In feces, the main metabolite was Rg1, but other deglycosylated metabolites, including F1 and protopanaxatriol, were also detected (Kim et al., 2013). Yang et al. (2009) identified 11 and nine metabolites together with Re in rat urine collected after intravenous (50 mg/kg, i.v.) and oral (100 mg/kg, p.o.) administration of Re, respectively. The metabolites included Rg1, Rg2, Rh1, and F1. Oral and intravenous doses of Re showed distinct metabolism patterns in the rat, but there were also certain characteristics in common. Deglycosylation was found to be the major metabolic pathway of Re in rats, indicating that a large part of Re was metabolized and transformed in the gastrointestinal tract to ginsenosides with more biological effects (Christensen, 2009). The Re may be metabolized into ginsenosides Rh1 and F1 by human intestinal microflora, and subsequently absorbed into the blood (Bae et al., 2005). After oral administration of 100 mg/kg Re to rats, Chen et al. (2009) detected six metabolites of Re in feces, including ginsenosides Rg_2_, Rh_1_, Rh1, F1, Rh1, and PPT. In general, Re may be hydrolyzed by gastric fluids to ginsenoside Rg2 that is then converted in the intestine into ginsenoside Rh1 by the elimination of rhamnose through intestinal bacteria. Intact Re also reaches the large intestine where it can be metabolized by bacteria into ginsenoside F1 and 20(S)-PPT *via* ginsenoside Rg1. Like intestinal bacteria, several food microorganisms produce specific forms of ginsenosides. (Chi and Ji, 2005) tested the biotransformation of Re by cell extracts from various food-grade edible microorganisms, and found Re was transformed into Rh_1_ *via* Rg_2_ by Bifidobacterium sp. Int57 and SJ32, Re was transformed into Rh_1_ *via* Rg_1_ by *Aspergillus niger* KCTC 6906, and Re was transformed into Rg_2_ by *A. usamii* var. *shirousamii* KCTC 6956.

### Elimination

Joo et al. (2010) found that Re was rapidly cleared from the bodies of male or female mice within 0.2 ± 0.03 and 0.5 ± 0.08 h, respectively, after intravenous administration. Chen et al. (1980) estimated that the half-life of Ren in rabbits, after intravenous administration, was about 0.83 h, and the elimination half-life of Re after i.p. injection could be measured from urine (1.165 h) but not plasma samples. In healthy volunteers, the half-life of Re after oral ingestion of Re tablets (200 mg/tablets, p.o.) was reported to be 1.82 ± 0.75 h (Liu et al., 2011). A randomized, double-blind, placebo-controlled trial reported that researchers were unable to detect Re in plasma of obese adults, even though the subjects were prescribed large daily oral doses of *P. ginseng* and Re for 30 days and ingested the last dose 30 min before collection of blood samples to assess Re concentrations. The absence of Re may be explained by the quick elimination of ginsenoside (Reeds et al., 2011). Pharmacokinetic studies of Re in rats and human volunteers were consistent with this statement. After intragastric (i.g.) administration of Banxia Xiexin Decoction in rats, plasma concentrations of Re at most time points were lower than the lower limit of quantification (Wang et al., 2008a). Pharmacokinetic studies of Re in rats and volunteers following i.v. administration of Shen Mai indicated that Re was quickly eliminated in the body, and that pharmacokinetic characteristics fitted the two-compartment model (Liu et al., 2005; Xia et al., 2008). Altogether, evidence from pharmacokinetic and metabolic studies of Re demonstrated that *1*) the absorption of Re was fast in the gastrointestinal tract; *2*) Re may be metabolized mainly into Rh1 and F1 by intestinal microflora before absorption into blood; and *3*) Re was quickly cleared from the body (Peng et al., 2012).

## Search Method

We included articles that were published from January 2000 to March 2021. Because more than 344 articles were found, we opted to focus on those specifically pertaining to new reports of the pharmacology, pharmacokinetics, and toxicology of Re. We searched four electronic databases, Google Scholar, NCBI, PubMed, and Web of Science, and compiled data according to the grade of evidence that was found. Systematic searches were performed in four electronic databases and the reference lists of most papers in the past 20 years were checked for further relevant publications. All articles containing original data on pharmacological activity, pharmacokinetics, and toxicology of Re were included. In addition, we only included studies written in English. Approximately 140 articles were used in the review process, across a variety of *in vitro* and *in vivo* studies, case reports, and randomized controlled trials.

## Pharmacological Effects of Re on Diabetes Mellitus (DM)

### Anti-DM Effects *In Vivo*

Attele et al. (2002) found that Re (20 mg/kg, i.p. for 12 days) had marked anti-hyperglycemic activities, with no effect on the body weight of C57BL/6J ob/ob mice. This finding suggests that Re has potential as an anti-diabetic agent. Re (10 mg/kg, i.p. for 12 days) significantly reduced fasting blood glucose levels and promoted glucose tolerance (GT) and systemic insulin sensitivity (IS) in ob/ob mice without affecting body weight (Xie et al., 2005a). These findings suggest Re may provide a therapeutic role in ameliorating GT and insulin resistance (IR) in patients with type 2 diabetes mellitus (T2DM). Administration of Re (0.2 mg/ml for 90 min) rapidly normalized IR and muscle glucose transport induced by high-fat diet (HFD) in the epitrochlearis and soleus muscles of rats (Han et al., 2012). Re may have specifically acted to ameliorate IR in muscles of rats because it failed to modify HFD-induced muscle glucose transport resistance following stimulation by contraction or hypoxia. Muscle contraction and hypoxia exert an insulin-like-stimulating effect on glucose transport. However, Re did not affect basal or insulin-stimulated muscle glucose transport in chow-fed rats. According to these animal studies, *P. ginseng* or ginsenoside appeared to improve oral GT and accelerate insulin-stimulated glucose disposal (Xie et al., 2004). The Re-induced improvement in IS may or may not be associated with weight loss. Therefore, it remains unclear whether the amelioration was due to weight loss or insulin-sensitizing traits. These studies demonstrated the association between the anti-hyperglycemic activity of Re and improved IS, whereas body weight was unaffected. The improvement may be attributed to the insulin-sensitizing properties of Re. Quan et al. (2012) studied the potential anti-glycemic role of Re in HFD-induced diabetes in mice. Administration of Re (20 mg/kg, i.g. for 3 weeks) markedly lowered BG and triglyceride levels and prevented hepatic steatosis in C57BL/6J mice on a HFD. The hypoglycemic effect was associated with suppression of hepatic gluconeogenesis, possibly associated with AMP-activated protein kinase (AMPK) activation. In rats on a HFD, Re (40 mg/kg, i.p. for 2 weeks, twice a day) improved IR by inhibiting c-Jun N-terminal kinase (JNK) and nuclear factor (NF)-kB activation (Zhang et al., 2008). Several studies have concluded that the anti-hyperglycemic effect of Re was primarily responsible for improved microvasculopathy or reduced cognitive impairment in HFD-induced diabetic mouse models. In such models, Re (20 mg/kg, i.g. for 8 weeks) exerted a protective and anti-angiopathy effect in DM, such as the initial stages of high-sucrose-HFD (HSHF)-induced diabetes, HSHF+alloxan monohydrate-induced Type 1 diabetes mellitus (T1DM), and HSHF+streptozotocin (STZ)-induced T2DM. Administration of Re reduced BG levels, regulated increasing insulin levels, improved lipid metabolism, and reduced endothelial cell dysfunction. The underlying mechanism was possibly associated with p38 mitogen-activated protein kinase (MAPK) activation, and extracellular signal-regulated kinase (ERK) 1/2 and JNK signaling (Shi et al., 2016). In addition, Re (20 mg/kg, i.g. for 2 weeks) had an anti-diabetic microvasculopathy effect, including protective actions against oxidative stress in the kidneys and eyes, and increased BG and lipid levels in rats with STZ-induced diabetes (Cho et al., 2006). In rats with STZ-induced T1DM, Re (40 mg/kg, i.g. for 8 weeks) improved diabetes-related cognitive decline while decreasing fasting BG levels, although it did not affect BG, which was associated with oxidative stress and inflammation (Liu et al., 2012). In mice, Re improved HFD-induced IR through amelioration of hyperglycemia by protecting the brain cholinergic and antioxidant systems (Kim et al., 2017). Specifically, Re (5, 10 and 20 mg/kg/d, i.g. for 4 weeks) improved diabetes-associated cognitive impairment, and was possibly associated with improvement of the anti-oxidant and cholinergic systems in brain tissue. In HFD-induced hyperglycemic C57BL/6 mice, Re played a positive role through amelioration of insulin tolerance and BG levels. Re possibly improved learning and memory disorders related to HFD-induced diabetes. As the major ginsenoside in the *P. ginseng* berry ethyl acetate fraction (blended with drinking water 20 and 50 mg/kg, p.o. for 4 weeks), Re ameliorated cognitive decline in a dose-dependent manner because of its cholinergic activity, and it decreased oxidative stress in mice with HFD-induced T2DM and behavioral deficiency (Park et al., 2015).

### Anti-DM Effects *In Vitro*

In 3T3-L1 adipocytes, Re (10 μM for 24 h) improved IR by inhibiting the inflammatory signaling cascade and activating the insulin signaling pathway (Zhang et al., 2008). Further results demonstrated that Re (1–10 μΜ for 0.5 h) increased glucose uptake in mature 3T3-L1 cells by significantly enhancing glucose transporter 4 (GLUT4) mRNA expression through the phosphoinositide 3-kinase (PI3K)-dependent pathway involving insulin receptor substrate-1 (IRS-1) in the glucose transport system cascade (Lee et al., 2011). Gao et al. (2013) demonstrated that Re (30, 60 μM for 5 days) reduced IR in adipocytes by directly enhancing the expression of peroxisome proliferator-activated receptor-γ (PPARγ)-2 and the corresponding AP2 genes, increasing adiponectin and IRS-1 expression, inhibiting inflammatory cytokine tumor nuclear factor-α (TNF-α) expression and production, and promoting GLUT4 translocation. The regulation of these factors facilitated adipocyte glucose uptake and disposal, although it failed to enhance GLUT4 expression. Another study found that Re (20 μM for 3 h) suppressed glucose generation in HepG2 cells, possibly by triggering the expression of the orphan nuclear receptor small heterodimer partner gene *via* AMPK activation (Quan et al., 2012). These results indicate that Re improved IR through reduction of lipotoxicity in the muscles and liver by enhancing adipocyte lipid storage capacity and promoting GLUT4 translocation to plasma membranes. Thus, Re compound regulation of insulin-stimulated glucose ingestion led to improved IR. Furthermore, Re (3 μM for 24 h) was proposed to exert anti-angiogenetic effects in diabetic retinopathy through the PI3K/Akt-mediated hypoxia-inducible factor-1-alpha (HIF-1α)/vascular endothelial growth factor (VEGF) signaling pathway in high-glucose-induced retinal endothelial RF/6A cells (Xie et al., 2020).

Overall, *in vivo* and *in vitro* data suggest four possible mechanisms underlying Re-induced improvement of diabetes and diabetes-related complications: *1*) regulation of insulin resistance and insulin secretion, *2*) modulation of glucose or lipid metabolism, *3*) modulation of inflammatory cytokines, and *4*) activation of oxidative stress.

## Pharmacological Effects of Re on Nervous Diseases

### Anti-Peripheral Nerve Injuries Effects *In Vivo* and *Vitro*

In rats with sciatic nerve crush injury, Re (2.0 mg/kg, i.p. for 4 weeks) promoted functional recovery, nerve regeneration, and proliferation of injured sciatic nerves. The Re compound promoted Schwann cell proliferation, differentiation, and migration during the course of peripheral neural repair after crush injury. This effect was possibly mediated by the regulation of ERK1/2 and JNK1/2 signaling pathways (Wang et al., 2015).

### Anti-Cerebral Ischemia Effects *In Vivo*

One study reported the anti-oxidant effects of Re (5, 10 and 20 mg/kg, i.g. for 1 week) in rats with cerebral ischemia-reperfusion (I/R) injury. The Re compound considerably increased membrane fluidity of brain mitochondria, activated anti-oxidative enzymes, and decreased lipid peroxidation products, including malondialdehyde (Zhou et al., 2006). Neuroprotective effects of Re (5, 10 and 20 mg/kg, i.g. for 1 week) against cerebral I/R injury in rats were associated with a reduction in malondialdehyde levels and mitochondrial swelling, leading to an increase in H^+^-ATPase activity (Chen et al., 2008).

### Anti-Neurotoxicity Effects *In Vivo*

Tu et al. (2017) reported that Re (20 mg/kg, i.p. for 3 days) attenuated convulsive behaviors, oxidative damage, pro-apoptotic potential and neuronal degeneration through the interleukin-6 (IL-6)-dependent PI3K/Akt signaling pathway in mice with trimethyltin-induced neurotoxicity. Treatment with Re (20 mg/kg, i.p. for 1 day) markedly decreased phencyclidine-induced neurotoxic alterations, including behavioral changes and mitochondrial dysfunction. These Re-mediated alterations were due to interactive modulation between glutathione peroxidase-1 (GPx-1) and NADPH oxidase in mice (Tran et al., 2017).

### Anti-Depression and Anti-Cognitive Dysfunction Effects

Administration of Re (50 mg/kg, i.p. for 10 days) before immobilization stress markedly improved body weight, serum corticosterone levels, behavioral alterations, and cognitive deficits in rats. These effects were mediated through modulation of the central noradrenergic system and hypothalamic corticotrophin-releasing factor in the brain (Lee B et al., 2012). Another study showed Re (20, 40 mg/kg, i.p. for 3 weeks) inhibited memory deficits induced by chronic restraint stress (Wang et al., 2021). The protective effects were related to anti-inflammatory and anti-oxidant activities of the Re compound, as well as positive regulation of brain-derived neurotrophic factor and plasticity-associated proteins in the hippocampus.

### Anti-Parkinson’s Disease (PD) Effects *In Vivo*

Administration of Re can effectively prevent onset of Alzheimer’s disease (AD) by improving the activity of dopamine (DA) neurons. One study found that Re (6.5, 13 and 26 mg/kg, i.g. for 13 days) prevented apoptosis of substantia nigra dopaminergic neurons induced by 1-methyl-4-phenyl-1,2,3,6-tetrahydropyridine in C57BL mice (Xu et al., 2005). The effect was mediated by reversing the abnormal expression of apoptosis regulatory proteins and inhibiting caspase-3 activation. Administration of Re (10, 20 mg/kg, i.p. for 2 weeks, twice a day) rescued methamphetamine-induced dopaminergic neurotoxicity. The effect was associated with potentiating oxidative burdens, compensative induction of GPx activity, mitochondrial dysfunction, pro-inflammatory changes, apoptotic cellular degeneration, and dopaminergic degeneration through inactivation of the protein kinase Cẟ (PKCδ) gene (Shin et al., 2014). Another study reported that Re (20 mg/kg, i.p. for 5 days, twice a day) protected methamphetamine-treated prodynorphin knockout mice against dopaminergic neurotoxicity through anti-oxidant, anti-inflammatory, and anti-apoptotic actions. The effects were facilitated by dynorphin-induced upregulation of the κ-opioid receptor, followed by substance P-mediated downregulation of the NK1 receptor (Dang et al., 2018).

### Anti-PD Effects *In Vitro*

Administration of Re (10 µM) and ginsenoside Rd (5 µM for 48 h) provided considerable neuroprotective effects on primary dopaminergic midbrain neurons treated with CCl_4_. The neuroprotective effects were in part due to the lowering of oxidative stress and alleviation of inflammatory responses (Zhang et al., 2016). In addition, Re treatment (50, 100 μM for 24 h) of SH-SY5Y cells rescued methamphetamine-induced mitochondrial burden (compensative induction of cytosolic and mitochondrial GPx activity, mitochondrial oxidative stress, mitochondrial dysfunction, and mitochondrial translocation of cleaved PKCδ, and pro-apoptosis through genetic inhibition of PKCδ) (Nam et al., 2015). Kim et al. (2012) investigated the actions of Re on mitochondrial dysfunction in a PD model. They found that Re (3 µM) targeted mitochondrial dysfunction and rescued the defective PINK1-Hsp90/LRPPRC-Hsp60-complex IV signaling axis of PINK1-null neurons by restoring nitric oxide (NO) levels. Co-treatment using Rd and Re (0.5, 1 μM for 24 h) protected SH-SY5Y cells against rotenone-induced toxicity by regulating molecular mechanisms that enhanced cell viability, including prevention of morphological changes, lowered oxidative stress, improved mitochondrial integrity and function, and inhibited apoptosis owing to oxidative stress (Gonzalez-Burgos et al., 2017). The anti-oxidant mechanism of Re in PD remains unclear. In SH-SY5Y cells treated with 6-hydroxydopamine to induce oxidative stress, the Re compound (25 µM for 9 h) mediated its anti-oxidant effect by upregulating a key antioxidant gene GPX4 *via* PI3K/Akt and ERK cascades (Lee et al., 2020).

### Anti-AD Effects *In Vivo*

Kai-Xin-San, a Chinese herbal formula, has been clinically administered at 3 g/kg (i.g. for 4 weeks) to treat animals with AD and neurosis. *P. ginseng*, a component of Kai-Xin-San, is known to enhance learning ability and memory. In addition, positive effects of Re and Rb1, the most abundant saponins, on learning ability and memory were reported (Wang et al., 2010). Amyloid β (Aβ) peptide plays an important role in AD. Zhou et al. reported that Re may interfere with AD progression by affecting the Aβ peptide (Zhou et al., 2020). Oral administration of Re (25 mg/kg, i.g. for 18 h) considerably reduced Aβ1-40 and Aβ1-42 levels in brains of Tg2576 mice (Chen et al., 2006). Furthermore, Li et al. (2018) demonstrated that Re (4 mg/kg, i.g. for 40 days) improved cognitive impairment, reduced Aβ accumulation, and restored biomarker levels, including amino acids, lecithin, and sphingolipids in the plasma of AD mice. Because of its effect on Aβ peptides, Re is increasingly considered a potential alternative drug for AD treatment. In addition, Re exhibits anti-dementia activity. The Re compound improved extracellular levels of DA and acetylcholine (Ach), particularly in the hippocampus. Also, Re (12.5, 25 and 50 mg/kg, s.c.) increased extracellular levels of DA and Ach in the medial prefrontal cortex (Shi et al., 2013).

### Anti-AD Effects *In Vitro*

Treatment with Re has been reported to improve AD by affecting Aβ peptide levels in several cell models. Liang et al. reported that Re markedly reduced the generation of Aβ proteins in N2a/APP695 cells. The effect of Re (50–100 μM for 24 h) on Aβ generation was mediated by PPARγ activation in combination with Aβ-site precursor protein-cleaving enzyme 1 inhibition (Cao et al., 2016). Treatment with Re (0.1–100 μM for 2 h) considerably reduced cell toxicity and increased the release of lactate dehydrogenase, thereby attenuating PC12 cell damage induced by Aβ peptides (Ji et al., 2006). In addition, Re (25 µM for 48 h) exhibited neuroprotective activity against neurotoxicity arising from Aβ25-35 in SH-SY5Y cells by reducing oxidative damage and neuronal cell apoptosis. The neuroprotective activity was associated with the activation of nuclear factor erythroid-2 associated factor 2/heme oxygenase-1 anti-oxidant response pathways and inhibition of reactive oxygen species (ROS)-dependent apoptosis signal-regulated kinase 1/JNK/Bax apoptosis pathways (Liu et al., 2019). Furthermore, Kim et al. demonstrated that Re (5 μg/ml for 48 h) effectively upregulated the expression of choline acetyltransferase and vesicular acetylcholine transporter, and Ach production in Neuro-2a cells, thus countering symptoms during AD progression (Kim J et al., 2014).

*In vivo* and *in vitro* data suggest six possible mechanisms of Re-mediated improvement of complications associated with nervous system diseases: *1*) regulation of central cholinergic pathways, *2*) modulation of the apoptotic signaling pathway, *3*) modulation of inflammatory responses, *4*) modulation of mitochondrial burden, *5*) regulation of anti-oxidant signaling pathways, and *6*) reduction of Aβ peptide accumulation and loss of midbrain DA neurons.

## Pharmacological Effects of Re on Inflammation

### Anti-Inflammatory Effects *In Vivo*

Treatment with Re considerably inhibited neutrophil infiltration in a model of skin inflammation arising from 12-O-tetradecanoylphorbol-13-acetate. It also improved paw and ear oedema, increased malondialdehyde levels in paw fluid during c-carrageenan-induced edema, and suppressed interleukin-1β (IL-1β) and TNF-α expression in lipopolysaccharide (LPS)-stimulated murine Raw 264.7 macrophages (Paul et al., 2012). Moreover, Re (1 mg/kg, i.v. for 15 min) suppressed the LPS-induced increase in body temperature, white blood cell count, and pro-inflammatory mediators (Su et al., 2015). In LPS-induced systemic inflammation, Re (10, 20 mg/kg, i.g. for 4 h) suppressed serum levels of IL-1β and TNF-α in mice. Similarly, in 2,4,6-trinitrobenzene sulfonic acid-induced colitic mice, Re (10, 20 mg/kg, i.g. for 3 days) suppressed the expression of IL-1β, TNF-α, cyclooxygenase-2, and inducible nitric oxide synthase, and the activation of transcription factor NF-κB. However, it enhanced the expression of anti-inflammatory cytokine IL-10, indicating that Re can suppress Th1 rather than Th2 cell activation (Lee I et al., 2012). Administration of Re (15 mg/kg, i.g. for 1 week) also prevented NF-κB activation and LPS-induced myocardial inflammation in mice. The action of Re in cardiac dysfunction involves both MAPK inhibition and preserved activation of estrogen receptors and the PI3K/Akt signaling pathway (Chen et al., 2016). Treatment with Re (6–50 mg/kg, p.o. for 2 h) produced strong and significant inhibitory actions against LPS-induced lung inflammation in mice, and decreased inflammatory cell infiltration into lung tissue. The effect was mediated by inhibiting the activation of MAPK and transcription factors NF-κB and c-Fos (Lee et al., 2018).

### Anti-Inflammatory Effects *In Vitro*

An *in vitro* investigation of the anti-inflammatory effects of Re (5, 10 μΜ for 30 min) in macrophages showed that it suppressed the expression of pro-inflammatory cytokines (TNF-α and IL-1β) and activation of transcription factor NF-κB by preventing the binding between LPS and toll-like receptor 4 (TLR4). However, Re did not suppress pro-inflammatory cytokines in peptidoglycan- or TNF-α-stimulated peritoneal macrophages (Lee J et al., 2012), highlighting its action in reducing inflammation by suppressing the LPS and TLR4 interaction in macrophages. Su et al. (2015) demonstrated that Re (50 μg/ml for 1 h) competed with LPS binding to the TLR4, and blocked the LPS-triggered signaling pathway in LPS-stimulated RAW264.7 cells. Extracellular Re was shown to compete with LPS binding to the TLR4, consistent with its role in the activation of extracellular TLR4 (Su et al., 2012). In addition, Wu et al. reported an anti-inflammatory role of Re (10–100 μΜ for 48 h) in LPS-induced activated N9 microglial cells. Re mediated its effects by inhibiting the generation of NO and TNF-α through downregulation of NF-κB activation (Wu et al., 2007). Treatment with Re (2 μg/ml for 24 h) reduced neuroinflammation by reducing the levels of inducible nitric oxide synthase and cyclooxygenase-2, and activating p38 MAPK in LPS-treated BV2 microglial cells (Lee K et al., 2012). Moreover, Quan H et al. (2019) reported that Re (10–40 μΜ for 24 h) inhibited LPS-induced TNF-α and IL-6 production in RAW264.7 cells, and reduced IL-6, NO, prostaglandin E2, and TNF-α secretion in primary rat hepatocytes *via* MAPK and NF-κB signaling pathways. Re is an effective component of Shen Fu, and was reported to exert anti-inflammatory effects by suppressing the NF-κB signaling pathway in TNF-α-stimulated EAhy926 cells (Li P et al., 2016). Incubation with Re (1.7 μg/ml for 24 h) decreased histamine secretion in human mast cells, and reduced IL-1α, IL-8, and IL-10 levels, and regulated T-cell-expressed and secreted protein secretion in A549 cells (Bae et al., 2012).

Altogether, *in vivo* and *in vitro* study data indicate that the possible mechanism of anti-inflammatory activities of Re involves NF-κB inactivation and reduced inflammatory cytokine release.

## Pharmacological Effects of Re on Cardiovascular Diseases (CVDs)

### Anti-Myocardial Injury Effects *In Vivo*

Kim et al. (2011) showed that Re improved ischemia/reperfusion (I/R) dysfunction by reversing the hemodynamic change (aortic flow, coronary flow, perfusion pressure, and cardiac output) and inhibiting the level of intracellular Ca^2+^ ([Ca^2+^]_i_). This study indicated that the anti-ischemic effect of Re was mediated by inhibiting an increase of [Ca^2+^]_i_. Additionally, Re prevented heart mitochondrial Ca^2+^ accumulation in I/R injury. In isolated single cardiomyocytes, Re suppressed the L-type Ca^2+^ current and strengthened the slowly activating delayed rectifier K^+^ current (I_Ks_). This may be the underlying mechanism that prevented mitochondrial Ca^2+^ overload (Bai et al., 2003; Bai et al., 2004).

A rat model showed that Re (20 mg/kg, i.g. for 15 days) provided an effective treatment for myocardial infraction arising from left anterior descending coronary artery ligation. Treatment with Re improved the parameters of myocardial injury by downregulating the expression of intercellular adhesion molecule-1 and inhibiting polymorphonuclear leukocyte infiltration (Jing et al., 2010; Li et al., 2013). In this research, Re was reported to exhibit a protective role in ischemia-induced myocardial injury by regulating calcium transport, preserving mitochondrial structure and function, enhancing anti-oxidant capacity, and recovering myocardial blood flow.

In addition, Re lowered myocardial injury and suppressed cardiac hypertrophy in experimental models with cardiac dysfunction. Lim et al. (2013) proposed that Re (100 μM, injected into the aortic line for 3 min) exerted beneficial effects on cardiac function in rats with I/R injury, considerably improved hemodynamic functions and left ventricular developed pressure, ameliorated electrocardiographic abnormalities, and decreased the production of TNF-α. Treatment with Re (5, 20 mg/kg, i.g. for 4 weeks) also reduced isoproterenol-induced myocardial fibrosis, increased heart weight and hydroxyproline content, and reduced heart failure. The molecular mechanisms underlying the protective role of Re were possibly related to regulation of the transforming growth factor-beta 1 (TGF-β1)/Smad3 pathway (Wang et al., 2019). In a rat model of myocardial injury, Re (135 mg/kg, i.g. for 4 weeks) preserved cardiac function and structure, reduced myocardial injury and stress, and decreased left ventricular fibrosis by regulating the AMPK/TGF-β1/Smad2/3 and FAK/PI3K/Akt signaling pathways (Yu et al., 2020). These findings suggest a possible therapeutic role for Re in suppressing ventricular remodeling and promoting postinfarction healing. Overall, Re restored blood supply quickly and also delayed detrimental ventricular remodeling during chronic myocardial infraction rehabilitation.

### Anti-Myocardial Injury Effects *In Vitro*

Wang et al. found that Re (200 μg/ml for 24 h) increased H9c2 cell viability after tertbutyl hydroperoxide treatment and reduced lactate dehydrogenase release and cell apoptosis (Wang et al., 2020). Treatment with Re (100 μM for 3 h) inhibited glucose deprivation-induced autophagy of H9c2 cardiac muscle cells, an effect which may be associated with the inhibition of autophagy, increase in cellular ATP content and viability, and alleviation of oxidative stress (Zhang et al., 2020). In addition, in the hypoxia/reoxygenation injury model, Re (100 µM for 21 h) increased HL-1 cell viability and ATP levels. The possible mechanism was that Re acted on the binding interface between HIF-1α and von Hippel-Lindau protein to prevent the binding of these proteins, thereby suppressing HIF-1α ubiquitination (Sun et al., 2020).

### Adjusting Electrophysiological Activities

Administration of Re (≥10 nM) effectively suppressed the electromechanical alternans of cardiomyocytes in cats and humans by increasing sarcoplasmic reticulum Ca^2+^-release channels, and thereby improving arrhythmia (Wang et al., 2008b). Furukawa et al. (2006) showed that Re (3 μM) increased I_Ks_, [Ca^2+^]_i_, activation of eNOS, and NO production through a c-Src/PI3K/Akt-dependent mechanism related to the non-genomic pathway of sex steroid receptors. Similarly, in vascular smooth muscle cells (VSMCs), Re non-genomically and dose dependently activated K_Ca_ currents and eNOS (EC_50_ = 4.1 ± 0.3 μM) through the c-Src/PI3-kinase/Akt pathway of the estrogen receptor (Nakaya et al., 2007). A study on human umbilical vein endothelial cells (HUVECs) revealed that Re augmented [Ca^2+^]_i_ and NO production in a dose-dependent manner (EC_50_ of 316 and 615 nM, respectively) (Leung et al., 2007). In human coronary artery endothelial cells, Re (1 μM) induced vasorelaxation by increasing small-conductance Ca^2+^-activated K^+^ (SK_Ca_) channel activity, stimulating NO production, and promoting vasodilation (Sukrittanon et al., 2014).

### Anti-Atherosclerosis Effects

Abnormal structure and function of VSMCs may result in the development and progression of arteriosclerosis. Enhanced proliferation and migration of VSMCs represent critical events during the course of atherosclerotic lesion development (Bennett et al., 2016). Gao et al. (2018) demonstrated that Re (25 or 50 mg/kg, i.g. for 2 weeks) inhibited VSMC proliferation by suppressing phenotypic modulation and inhibiting vascular neointimal hyperplasia in balloon-injured rats through the eNOS/NO/cyclic guanosine monophosphate (cGMP) pathway. Re improved platelet-derived growth factor-BB-induced VSMC proliferation through G_0_/G_1_ cell cycle arrest, which was associated with eNOS/NO/cGMP pathway activation (Gao et al., 2019).

In contrast, endothelial cells provide an interface between circulating blood in the lumen and other vessel walls. Endothelial cells exhibit great sensitivity and vulnerability to toxic substances circulating in blood vessels. Endothelial dysfunction is an important contributor to the pathobiology of atherosclerosis (Gimbrone and García-Cardeña, 2016). Huang et al. (2016) found that Re (4, 16, and 64 μmol/L for 24 h) attenuated oxidative damage in H_2_O_2_-induced HUVECs and increased the production of NO and eNOS, superoxide dismutase (SOD), and GPx activities. The protective effects were associated with an oxidative stress response, protein synthesis and mitochondrial function. In addition, Yang et al. demonstrated that Re (120 μg/ml for 12 h) improved oxidized low-density lipoprotein-induced endothelial cell apoptosis. The effect was possibly elicited through regulation of oxidative stress, inhibition of inflammatory mediators, and recovery of balanced pro- and anti-apoptotic protein expression *via* p38/MAPK/NF-κB and PI3K/Akt/NF-κB pathways. These pathways may be regulated by the lectin-like oxidized low-density lipoprotein receptor-1, NADPH oxidase, and estrogen receptor α (Yang et al., 2018). Therefore, Re is a potential anti-oxidant that may be used to protect HUVECs from damage by oxidative stress through the anti-oxidant defense system. The Re compound also inhibited VSMC proliferation, attenuated endothelial dysfunction, and possibly promoted NO production, thereby reducing atherosclerosis.

### Promoting Angiogenesis

Re is a pro-angiogenic compound with high stability that upregulates *in vitro* proliferation, migration, chemo-invasion, and tube formation of HUVECs. It also affects *ex vivo* aortic sprouting and *in vivo* neovascularization. *In vitro* results revealed that Re (10–30 μg/ml for 48 h) dose dependently enhanced the proliferation, migration, and tube formation of HUVECs (Huang et al., 2005). Additionally, extracellular matrix incorporating Re (70 μg for 1 week and 1 month) induced angiogenesis and enhanced tissue regeneration by increasing neocapillary density and tissue hemoglobin in a rat model (Yu et al., 2007). These findings indicate that Re can serve as an angiogenic agent to accelerate tissue regeneration.

In summary, *in vivo* and *in vitro* reports suggest five possible mechanisms by which Re may improve the cardiovascular system: *1*) attenuation of myocardial ischemia, *2*) inhibition of [Ca^2+^]_i_ and activation of I_Ks_, *3*) increased NO production, *4*) reduced cardiomyocyte apoptosis autophagy, and *5*) the regulation of oxidative stress.

## Pharmacological Effects of Re on Cancer

### Reduction In Side Effects of Chemotherapy

A combination of Re and cisplatin increased the survival rate of LLC-PK1 cells by 21.4%. However, the renoprotective effects of Re were weaker than that of Maillard reaction products in Re-leucine/serine and glucose-leucine mixtures. Moreover, Maillard reaction products reduced cisplatin-induced oxidative kidney damage by increasing 1,1-diphenyl-2 picrylhydrazyl radical-scavenging activity and decreasing the expression of cleaved caspase-3 protein in rats (Lee W et al., 2012; Kim M et al., 2014). (Wang et al., 2008c) found that Re (25 mg/kg, i.g. for 10 days) considerably suppressed acute kidney injury induced by cisplatin in mice, by inhibiting the oxidative stress damage, inflammatory response, and apoptosis. Re (5, 10 mg/kg, i.p. for 1 week) also improved cyclophosphamide-induced myelosuppression, alleviated clinical symptoms of myelosuppression, and promoted recovery of bone marrow hematopoietic functions. The possible mechanisms involved the regulation of hematopoiesis-related cytokine levels, promotion of cellular entry to the normal cell cycle, and improvement of bone marrow nucleated cell apoptosis-related protein expression (Han et al., 2019).

### Anti-Cancer Effects *In Vitro*

One mg/mL of American *P. ginseng* berry extract (containing 15.1 mg/g of Re for 72 h) exhibited strong anti-proliferative effects and triggered morphological alterations in SW480 human colorectal cancer cells (Xie et al., 2011). Re-carbon dots (0.5 mg/ml for 4 h) inhibited cancer cell proliferation (A375, HepG2, and MCF-7 cells) through the ROS-mediated pathway. However, the inhibitory effect on A375 cells was higher than that on other cells. Re induced apoptosis *via* the ROS- and caspase-mediated pathways (Yao et al., 2018). These findings demonstrate that Re can be used as a potential anti-cancer adjuvant for preventing and treating various cancers.

Altogether, *in vivo* and *in vitro* data show three possible mechanisms underlying the anti-cancer activities of Re: *1*) inhibition of cell proliferation, *2*) induction of cell apoptosis and *3*) modulation of oxidative damage.

## Pharmacological Effects of Re on Other Diseases

### Anti-Viral and Enhancement of Immune Response

Song et al. (2014) demonstrated that Re (100 μg/ml for 48 h) had potential therapeutic efficacy in CVB3 and HRV3 infections in HeLa and Vero cells, respectively. Su et al. (2014) showed that co-administration of Re (5.0 mg/kg. s.c. for 3 weeks) with the rabies virus vaccine remarkably increased the serum antibody response in mice. Other studies have shown that co-administration of Re (50 μg, s.c. for 3 weeks) with inactivated influenza virus A/Fujian/411/2002 (H3N2) markedly amplified serum-specific antibody responses (IgG, IgG1, IgG2a, and IgG2b), hemagglutination inhibition titers, lymphocyte proliferation responses, and IL-5 and IFN-γ production (Song et al., 2010). Chan et al. (2011) reported that Re (50 μΜ for 16 h) protected HUVECs from H9N2/G1 influenza virus-induced apoptosis. CD4^+^ T cells are important immune cells in the human immune system. Son et al. found that Re (10, 20 and 40 μg/ml for 24 h) enhanced the viability of activated CD4^+^ T cells by downregulating IFN-γ production, which interfered with autophagy by reducing immunity-associated GTPase family proteins (Son et al., 2010). Re also enhanced the expression of Th1-type-related and Th2-type-related cytokines (Su et al., 2014). Administration of Re (10, 25 and 50 μg, s.c. for 2 weeks) had considerable adjuvant effects on specific antibody and cellular responses in ovalbumin-immunized mice, affecting the immune system favoring Th1- or Th2-type responses, as shown by enhanced titers of IgG1 and IgG2b isotypes (Sun et al., 2006). These results indicated Re-mediated activation of Th1 and Th2 immune responses in mouse models. Therefore, these studies indicate that Re can enhance the host immune system as a vaccine adjuvant.

### Anti-Osteoporotic Effects

An optimal balance of osteoblasts and osteoclasts is crucial for bone remodeling. Impaired bone homeostasis potentially causes bone disease, such as bone fracture and osteoporosis (Feng and McDonald, 2011). It was demonstrated that Re had dual effects promoting osteoblast differentiation and inhibiting osteoclast differentiation. This research showed that Re (2.5, 5 and 10 μM for 48 h) dose dependently inhibited osteoclast differentiation and decreased nuclear factor of activated T cell cytoplasmic 1 and tartrate-resistant acid phosphatase mRNA levels, which are osteoclast differentiation markers. These effects were elicited by blocking the ERK signaling pathway in bone marrow-derived macrophages stimulated with the receptor activator of NF-κB ligand. Osteoclast generation in zebrafish scales was inhibited by Re (10 μM for 5 weeks), shown by reduced expression of osteoclast marker genes tartrate-resistant acid phosphatase and cathepsin K (Park et al., 2016). Kim et al. (2016) found that Re affected the differentiation and mineralization of osteoblasts both *in vitro* and *in vivo* models. Treatment with Re (50 µM for 5 weeks) promoted the expression of osteoblastic markers, including alkaline phosphatase activity, and mRNA levels of alkaline phosphatase, type 1 collagen, and osteocalcin in mouse osteoblast precursor MC3T3-E1 cells. Moreover, Re amplified the mineralization of osteoblasts in mouse MC3T3-E1 cells and zebrafish scales.

### Improving Skin Barrier Function

Treatment with Re (5, 12, and 30 μM for 0.5 h) provided potential anti-photo-ageing activity in HaCaT keratinocytes under UVB radiation. This activity was possibly elicited through downregulation of UVB-induced intracellular ROS formation, production and secretion of pro-matrix metalloproteinase-2 and -9, and upregulation of total GPx levels and SOD activity (Shin et al., 2018). In addition, Oh et al. (2016) found that Re (5, 12 and 30 μM for 1 h) improved skin barrier functions, shown by enhanced cornified cell envelope formation, filaggrin levels and caspase-14 activity in HaCaT keratinocytes. Furthermore, Re (5, 12 and 30 μM for 24 h) demonstrated anti-oxidative activity through the upregulation of anti-oxidant components including total GPx and SOD under normal conditions. Re also prevented oxidative stress in HaCaT keratinocytes (Lim et al., 2016).

### Anti-Oxidative Effects

Re (0.05, 0.1 and 0.5 mg/ml for 2 h) protected chick cardiomyocytes from exogenous H_2_O_2_- and endogenous antimycin A-induced oxidative stress. The underlying mechanism for this protective effect involved scavenging of H_2_O_2_ and hydroxyl radicals. However, in an electron spin resonance spectroscopy study, Re did not reduce the 1,1-diphenyl-2 picrylhydrazyl-induced electron spin resonance signals for xanthine oxidase or H_2_O_2_ (Xie et al., 2006). Therefore, direct scavenging of free radicals was impossible through a single anti-oxidation pathway *in vivo*. The anti-oxidative effects of Re were achieved through activation or enhancement of the intracellular anti-oxidant system.

### Regulating Cholesterol Metabolism

Kawase et al. (2014) reported that Re (0.1–1 μM for 24 h) exerted a positive effect on cholesterol metabolism, increasing the expression level of sterol 12a-hydroxylase mRNA in rat primary hepatocytes, thereby facilitating cholic acid generation within bile acids.

### Alleviating Allergic Response

Jang et al. (2012) reported that Re (25 mg/kg, p.o. for 6 h) potently alleviated scratching behavior in mice with histamine-induced itch, by inhibiting the activation of transcription factors (NF-κB and c-jun), as well as the expression of IL-4 and TNF-α.

### Increasing Sperm Motility

Zhang et al. (2006) demonstrated that Re (100 μM for 2 h) improved sperm motility from fertile and asthenozoospermic infertile human subjects by enhancing NOS activity to promote endogenous NO generation.

### Restoring Erectile Dysfunction

The Re-enriched fraction (containing 109.0 mg/g of Re, 54.5 mg/kg, i.g. for 5 weeks) of *P. ginseng* berries effectively restored ethanol-induced erectile dysfunction in male rats through the NO-cGMP pathway (Pyo et al., 2016).

### Promoting Cyclic Growth of Hair Follicles

(Li Z et al., 2016) reported that topical treatment (5 mg/day, topical application on the back for 9 weeks) with Re markedly triggered hair shaft growth through selective suppression of hair growth phase transition-associated signaling pathways and TGF-β signaling cascades in nude mice.

### Reducing Gastrointestinal Motility Dysfunction

Re-mediated bidirectional regulation is dependent on the jejunal contractile status and requires the co-existence of the enteric nervous system, Ca^2+^, and Cajal interstitial cells. The stimulatory role of Re (10 μM) on jejunal contractility of rat isolated jejunal segments was associated with cholinergic stimulation, whereas its inhibitory role was associated with adrenergic activation and the NO-relaxing mechanism (Xiong et al., 2014). In addition, Re (40 μΜ) inhibited pacemaker potentials through ATP-sensitive K^+^ channels and the cGMP/NO-dependent pathway in cultured Cajal interstitial cells obtained from the small intestine of mice (Hong et al., 2015). Re (20, 100 mg/kg, i.g. for 30 min) ameliorated acute gastric mucosal lesions induced by compound 48/80, possibly by triggering mucus secretion and decreasing neutrophil infiltration, inflammation, and oxidative stress in gastric mucosa (Lee et al., 2014).

## Toxicology of Re

An acute toxicity study in mice treated with *P. ginseng* extract found LD_50_ values of 10–30 g/kg (Brekhman and Dardymov, 1969). Chronic treatment of mice and rats with *P. ginseng* extract (5 g/kg, p.o. for 2 years) produced almost no toxic effects, and the appearance, behavior, weight, and various physiological/histological indexes were within reasonable ranges (National Toxicology Program, 2011). Likewise, (Lu et al., 2012) found that the LD_50_ of Re was 5.0 g/kg in mice. In addition, in a chronic toxicity study, male and female SD rats treated with 375 mg/kg/day (orally) Re for 26 weeks, well below the typically non-toxic range (5–15 g/kg) of chemical substances (Hayes and Loomis, 1996), did not exhibit death, adverse reactions, and organ abnormalities (Lu et al., 2012).

### Reproductive and Developmental Toxicology

*In vitro* rat embryo cultures found that 50 μg/ml Re induced severe developmental delay and significantly reduced the morphological scores of all organ systems, but was not teratogenic to specific organ systems (Chan et al., 2004). However, *in vitro* embryotoxicity may not reflect the human situation, and limited information about the blood concentration of Re in humans was available from the medical literature. Further investigations are necessary to evaluate the pharmacokinetics and placental transfer of ginsenosides in humans.

### Carcinogenicity

No chronic carcinogenicity studies of Re in experimental animals have been found in the literature.

### Adverse Effects

Several studies reported that some patients had vaginal bleeding and breast pain owing to the estrogen-like effects of *P. ginseng* (Palmer et al., 1978; Greenspan, 1983; Kabalak et al., 2004). The Re compound has an estrogen-like effect (Bae et al., 2005), and may have similar side effects, but these have not been reported in the literature.

## Conclusions

Previous studies have shown that Re is abundant in the leaves, berries, flower buds, and roots of *P. ginseng* plants (Gao et al., 2018), in which the Re compound accounts for more than 30% of the total ginsenoside content (Wang et al., 2008a). Its pharmaco-economical merits support its use in natural supplements or drug formulations. Although Re is a relatively abundant ginsenoside with well-known pharmacological effects, to date, little is known about its pharmacokinetic profiles. Several studies have shown that because of its low bioavailability after oral absorption, its therapeutic effect is poor. Therefore, in-depth pharmacokinetic studies of Re should be performed to examine the presence of active metabolites. The identification of these metabolites may provide pivotal information regarding the bioactive forms of the ginsenoside Re and its pharmacological mechanisms. The potential therapeutic effect of Re may be improved by modifying the mode of administration or chemical structure. Structural changes in ginsenoside after heat processing may be strongly related to improvement in biological activity. After heat processing, Re demonstrated improved therapeutic efficacy, including anti-oxidant and anti-cancer activities (Lee B et al., 2012). Therefore, this area could be a new focus for future research.

Studies have shown that the Re compound has therapeutic efficacy on DM, neurological disorders, inflammatory responses, CVD and cancer. Moreover, multiple studies had shown a role for Re in treating hyperglycemia and hyperlipidemia in models of diabetes. Literature searches indicated that Re-induced improvement in the above-mentioned conditions were associated with anti-oxidant and anti-inflammatory properties, part of which were elicited through suppression of the p38-MAPK-mediated signaling pathway or activation of the PI3K/Akt and NF-κB signaling pathways. The anti-oxidant effect of Re was achieved by activating or enhancing the intracellular anti-oxidant system.

**FIGURE 1:**
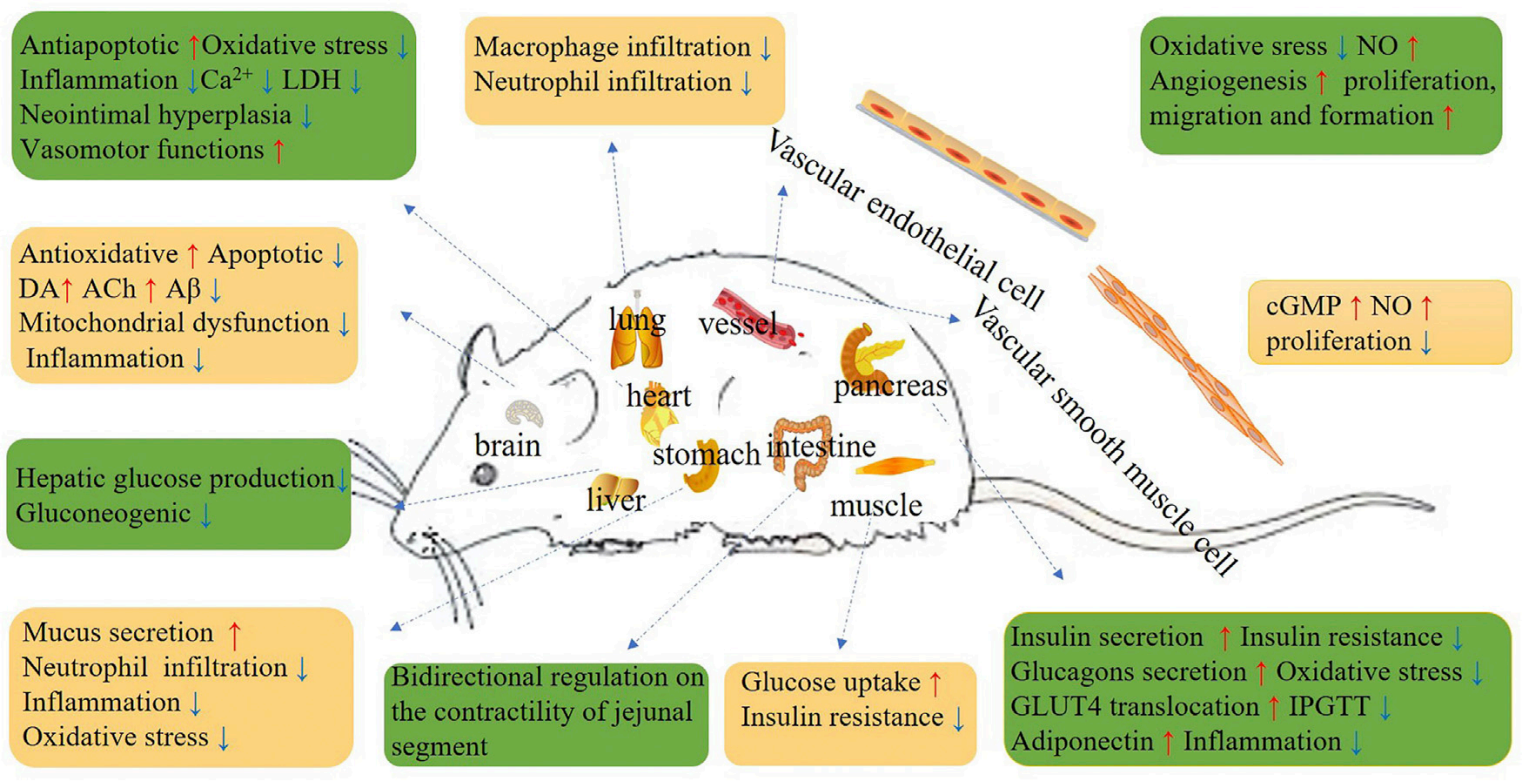
Schematic diagram depicting the beneficial effects of Re.

In conclusion, the beneficial properties of Re for DM, nervous system diseases, inflammatory responses, CVD, cancers, viral infections, oxidative stress, cholesterol metabolism, allergic and immune responses (Figure 1) indicate its potential as a novel treatment agent, but these properties need to be verified by future clinical experiments.

## Glossary

4-HNE: 4-hydroxynonenal
6-HODA: 6-hydroxydopamine
Aβ: amyloid β
ACh: acetylcholine
AChE: acetylcholinesterase
AD: alzheimer disease
ALP: alkalin phosphatase
AM: alloxan monohydrate
AMPK: AMP-activated protein kinase
ASK1: apoptosis signal-regulated kinase-1
ATA: antimycin A
AUC: area under curve
BDNF: brain-derived neurotrophic factor
BACE1: Aβ-site precursor protein-cleaving enzyme 1
bFGF: basic fibroblast growth factor
BG: blood glucose
BMNC: bone marrow nucleated cell
BNP: brain natriuretic peptide
BUN: blood urea nitrogen
BW: body weight
[Ca^2+^]_i_: intracellular Ca^2+^ homeostasis
cGMP: cyclic guanosine monophosphate
c-FOS-IR: c-Fos-immunoreactivity
CAT: catalase
ChAT: choline acetyltransferase
colla1: type 1 collagen
CRE: creatinine
CRS: chronic restraint stress
CTX: cyclophosphamide
CVDs: cardiovascular diseases
CYP8B1: sterol 12a-hydroxylase
CP: constipation-prominent
C_max_: peak concentration
CL: clearance
SFI: Shenfu Injection
DA: dopamine
DBP: diastolic blood pressure
DII: dexamethasone + 3-isobutyl-1-methylxanthine + insulin
DM: diabetes mellitus
DPPH: 1,1-diphenyl-2 picrylhydrazyl
DP: diarrhea-prominent
EDV: end-diastolic volume
ERs: oestrogen receptors
ERK: extracellular signal-regulated kinase
ESV: end-systolic volume
f: fraction excreted unchanged in the urine
FAS: fatty acid synthase
FBG: fasting blood glucose
GD: glucose deprivation
GOT: glutamic oxaloacetic transaminase
GPL: Glucose + sodium pyruvate + sodium lactate
GPT: glutamic pyruvic transaminase
GSH: glutathione
GT: glucose tolerance
GLUT4: glucose transporter 4
GPx: glutathione peroxidase
GGSQ: Gegen-Sanqi
HDL-C: high density lipoprotein cholesterol
HFD: high-fat diet
HGB: hemoglobin
HIF-1α: hypoxia-inducible factor-1-alpha
HO-1: heme oxygenase-1
HSHF: high-sucrose-HFD
HUVEC: human umbilical vein endothelial cell
I_Ca,L_: L-type Ca^2+^ current
iNOS: nitric oxide synthase
JNK: c-Jun N-terminal kinase
ICAM-1: intercellular adhesion molecule-1
IFN-γ: interferon-γ
IL-1β: interleukin-1β
IL-6: interleukin-6
Lp-α: lipoprotein α
IPGTT: intraperitoneal glucose tolerance test
I/R: ischamia/reperfusion
IR: insulin resistance
IRS-1: insulin receptor substrate
LRPPRC: leucine-rich pentatricopeptide repeat-containing
IS: insulin sensitivity
IVS: interventricular septum
IVSTd: IVS end-diastolic thickness
ICP: intracavernous pressure
LADCA: left anterior descending coronary artery
LC3B-2: microtubule-associated protein 1A/1B-light chain 3
LDH: lactate dehydrogenase
LDL-C: low density lipoprotein cholesterol
LKB1: liver kinase B1
LPC: lysophosphatidylcholine
LPS: lipopolysaccharide
LV: left ventricular
LVD: LV dimension
LVDd: LV end-diastolic dimension
ILVDs: LV endsystolic dimension
LVPWTs: LV posteriorend-systolic thickness
LVPWTd: LV posteriorend-diastolic thickness
LVEDP: Left ventricular end diastolic pressure
MA: methamphetamine
MAPK: mitogen-activated protein kinase
MCAO: middle cerebral artery occlusion
MDA: malondialdehyde
MI: myocardial infraction
MMP: metalloproteinase
MPO: myeloperoxidase
MPTP: 1-methyl-4-phenyl-1,2,3,6-tetrahydropyridine
MRPs: maillard reaction products
MLCK: myosin light chain kinase
MRT: mean residence time
NF-kB: nuclear factor-kB
NLRP3: NOD‐like receptor protein 3
NK1: neurokinin1
NO: nitric oxide
Nrf2: nuclear factor erythroid-2 associated factor 2
ox-LDL: oxidized low density lipoprotein
OVA: ovalbumin
QXSBP: QixueShuang bu Prescription
p75: nerve growth factor receptor
PCNA: proliferating cell nuclear antigen
PCP: phencyclidine
PD: parkinson disease
PEPCK: phosphoenolpyruvate carboxykinase
PG: peptidoglycan
PGE2: prostaglandin E2
PHOX: NADPH oxidase
PI3K: phosphoinositide3-kinase
PMN: polymorphonuclear leukocyte
PKCδ: protein kinase Cδ
PPARγ: peroxisome proliferator-activated receptor-γ
PSD95: postsynaptic density 95
RC: renal clearance
RANKL: receptor activator of NF-κB ligand
RANTES: T-cell-expressed and secreted
RBCs: red blood cells
RIS: repeated immobilization stress
RV: rabies virus vaccine
SCD1: stearoyl-CoA desaturase-1
SHP: small heterodimer partner
SNC: sciatic nerve crush injury
SOD: superoxide dismutase
SREBP: sterol regulatory element-binding protein
STZ: streptozotocin
SYP: synaptophysin
T1DM: type 1 diabetes
T2DM: type 2 diabetes
tBHP: tertbutyl hydroperoxide
TC: total cholesterol
TG: triglyceride
TH: tyrosine hydroxylase
TLR4: toll-like receptor 4
TMT: trimethyltin
TNBS: 2,4,6-trinitrobenzene sulfonic acid
TNF-α: tumour nuclear factor-α
TPA: 12-O-tetradecanoylphorbol-13-acetate
TPO: thrombopoietin
TRAP: tartrate-resistant acid phosphatase
TGF-β: transforming growth factor-β
TRARS: thiobarbituric acid reactive substances
T_1/2_: elimination half-life
T_max_: time for maximum concentration
VAchT: vesicular acetylcholine transporter
VEGF: vascular endothelial growth factor
VHL: von Hippel-Lindau
VSTs: IVS end-systolic thickness
V_d_: volume of distribution
WBC: white blood cell
XO: xanthine oxidase
XSTDT: Xue saitong dispersible table
